# Supplementary material for: Patatin-Related Phospholipase AtpPLAIIIα Affects Lignification of Xylem in Arabidopsis and Hybrid Poplars
Source: Plants (Basel). 2020 Apr 3;9(4):451. doi: 10.3390/plants9040451 (PMC7238252; doi:10.3390/plants9040451)
Supplement: Supplementary file 1 [file plants-09-00451-s001.zip › supplementary material/plants-745372-supplementary Figures.pdf]

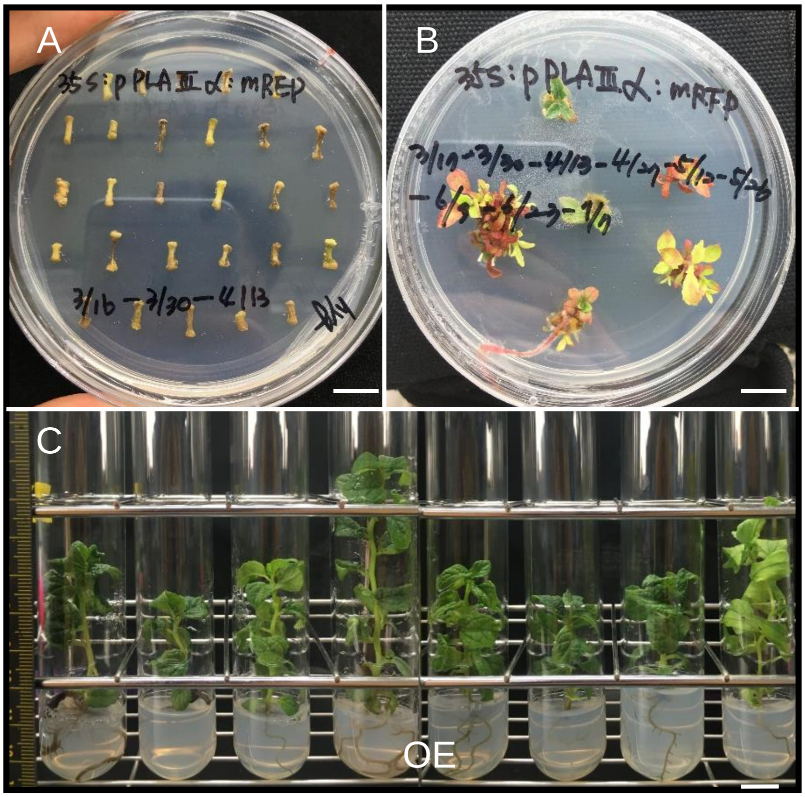

D

Total stem segment (number)	76
Callus inducing stem segments (number)	37
rate (%)	48 %
Total Callus (number)	37
Shoot inducing callus (number)	17
rate (%)	45 %
Total Shoot (number)	17
Regenerated whole plants (number)	8
rate (%)	47 %
Total stem segments (number)	76
Regenerated whole plants (number)	8
**Whole plant regeneration rate (%)**	**10 %**

Figure S1. Poplar transformation and the procedure of whole plant regeneration. (a) Callus formation from stem segments. (b) Shoot induction from callus. (c) Whole plant regeneration from selected transgenic shoot. (d) Recorded rates of callus, shoot induction, and whole plant regeneration. Transgenic plants were obtained with a rate of 10%. Bars = 1 cm.

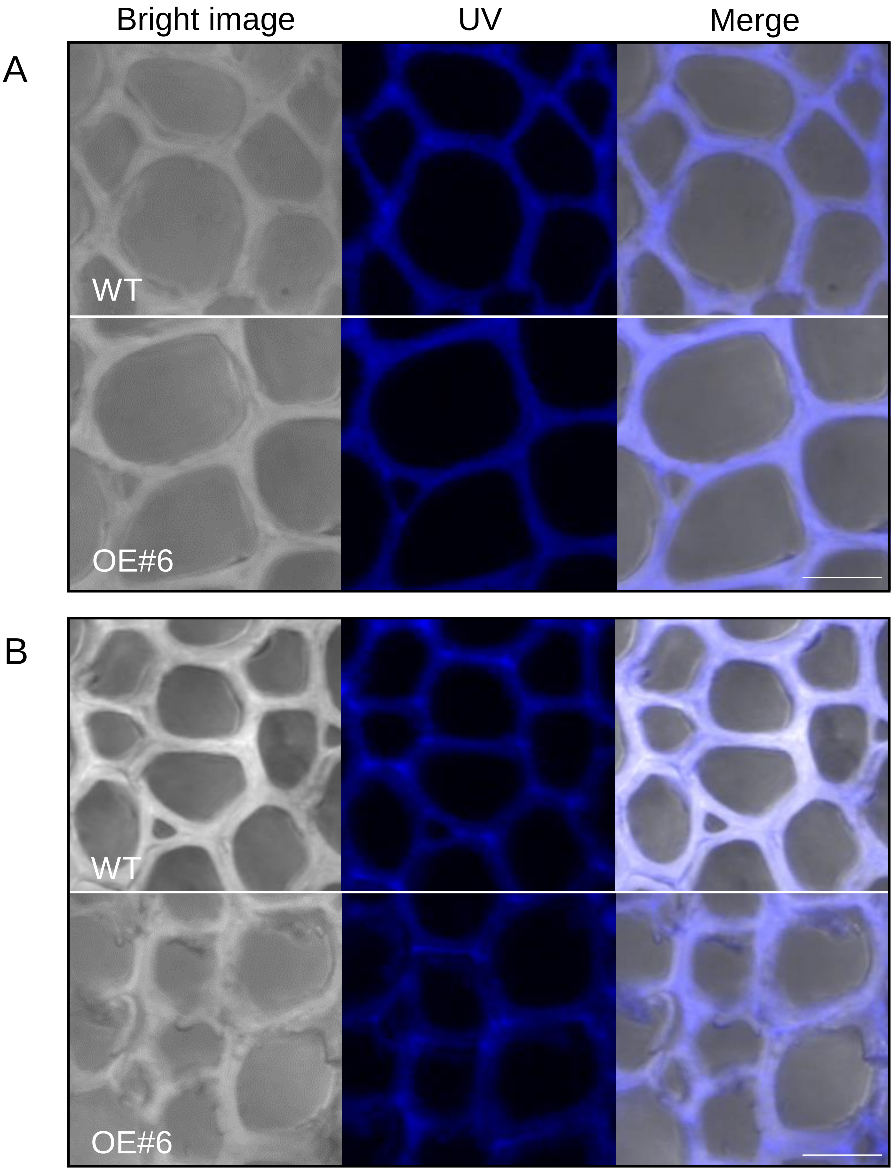

Figure S2. UV autofluorescence of xylem cells of WT and *AtPLAIIIα*OE. (A) Outer and (B) inner xylem layer structure image. Bars = 10 μm.
