# Supplementary material for: Patatin-Related Phospholipase AtpPLAIIIα Affects Lignification of Xylem in Arabidopsis and Hybrid Poplars
Source: Plants (Basel). 2020 Apr 3;9(4):451. doi: 10.3390/plants9040451 (PMC7238252; doi:10.3390/plants9040451)
Supplement: Supplementary file 1 [file plants-09-00451-s001.zip › supplementary material/Table S1.docx]

**TABLE S1.** List of DNA primers used in this study for confirmation of gene insertion and qRT-PCR

| Gene | Accession No. | Annotation | Primers used (5’-3’) |
| --- | --- | --- | --- |
| *AtpPLAIIIα* | At2g39220 | AtpPLAIIIα-F | GACGGATATGCAAGAACCGAGCAT |
|  |  | AtpPLAIIIα-R | TTCGAACCTGACCCACCCGAACCA |
| *AtpPLAIIIα* | At2g39220 | AtpPLAIIIα-SalI-5 | TCGTCGACATGTTAACTACGATGCAA |
|  |  | AtpPLAIIIα-AvrII-3 | GGCCTAGGAAACATACAATCAATATC |
| *AtActin* | At5g09810 | Atactin-2F | GTGTGTCTTGTCTTATCTGGTTCG |
|  |  | Atactin-2R | AATAGCTGCATTGTCACCCGATACT |
| *AtMYB58* | At1g16490 | AtMYB58-F | CCAGAGAACAGAGCTCTTCAAGAG |
|  |  | AtMYB58-R | ATGTATGAGGAGCTCGTAACTCTC |
| *AtMYB63* | At1g79180 | AtMYB63-F | GAACAGCTCAGGCTCAAGAGCAAC |
|  |  | AtMYB63-R | ATGTATCATGAGCTCGTAGTTCTT |
| *AtPAL1* | At2g37040 | AtPAL1-F | CAACGTACCCGTTGATTCAG |
|  |  | AtPAL1-R | TCCTCGAAAGCTCCAATCTT |
| *At4CL* | At1g51680 | At4CL-F | TCAACCCGGTGAGATTTGTA |
|  |  | At4CL-R | TCGTCATCGATCAATCCAAT |
| *AtHCT* | At5g48930 | AtHCT-F | CTCTTTCCAAAGCCCTTGTC |
|  |  | AtHCT-R | TCAGCCACAACGAAGAGAAC |
| *AtCOMT1* | At5g54160 | AtCOMT1-F | GTCGATTGCATTATGTTGGC |
|  |  | AtCOMT1-R | AGCCTGATGCTTTGGCTAAT |
| *AtCCR1* | At1g15950 | AtCCR1-F | TCCAGATGATCCGAAGAACA |
|  |  | AtCCR1-R | CGCCTTAAGAGCCTCGTAGT |
| *AtF5H2* | At5g04330 | AtF5H2-F | ATCATGGATGTGATGTTCGG |
|  |  | AtF5H2-R | ATCTCGGTTAGCACCCATTC |
| *AtACS11* | At4g08040 | AtACS11-F | GGTTTGTTCTGTTGGGTTGAC |
|  |  | AtACS11-R | AATGACACGATGAGCCTGG |
| *AtACO2* | At1g62380 | AtACO2-F | CGGGAAGTATAAGAGTGTGCTG |
|  |  | AtACO2-R | GGGTACTCGGAATCTTTCTCG |
| *AtACO4* | At1g05010 | AtACO4-F | TTTCTACCTCAAGCACCTTCC |
|  |  | AtACO4-R | CGGCGAAGTCTTTCATTAACG |
| *PtActin2* | Potri.019G01040.1 | PtActin2-F | ACCCTCCAATCCAGACACTG |
|  |  | PtActin2-R | TTGCTGACCGTATGAGCAAG |
| *PtPAL1* | Potri.006G126800.1 | PtPAL1-F | TTGACTTGAGGCATTTGGAG |
|  |  | PtPAL1-R | CAATGGATAGGTAGCACTGC |
| *PtC4H1* | Potri.013G157900.1 | PtC4H1-F | ACTCTGGGACGTTTGGTACA |
|  |  | PtC4H1-R | GCTTCATAGATTTACAGTGA |
| *Pt4CL1* | Potri.001G036900.1 | Pt4CL1-F | TAGTGAAATCAGAAAAGTCT |
|  |  | Pt4CL1-R | CGCAAGTATTAAAGAAATAA |
| *PtHCT1* | Potri.003G183900.1 | PtHCT1-F | ATCAGCATGTAAGGCACGCGG |
|  |  | PtHCT1-R | TGCCAAAGTAACCAGGTGGAAGCGT |
| *PtC3H3* | Potri.006G033300.1 | PtC3H3-F | GAGGTTCCTGGAGGAGGATG |
|  |  | PtC3H3-R | GGAGTCGTCATGTAAGTGAC |
| *PtCCOAOMT1* | Potri.009G099800.1 | PtCCOAOMT1-F | CAAGAGGTTGATTGAGCTTG |
|  |  | PtCCOAOMT1-R | GGTCAGCAGCAAGTGCCTTG |
| *PtCCR2* | Potri.003G181400.1 | PtCCR2-F | CTGTTCAAGCTTATGTGCATG |
|  |  | PtCCR2-R | GTGGAGAACGCTCTCAGAGC |
| *PtCOMT2* | Potri.012G006400.1 | PtCOMT2-F | CATGAAGTGGATATGCCATG |
|  |  | PtCOMT2-R | GTTGAATGCACAGCACATTAC |
| *PtCAD1* | Potri.009G095800.1 | PtCAD1-F | CAAGCTGATCTTGATGGGTG |
|  |  | PtCAD1-R | CGAATCTATATCTCACATC |
| *PtoMYB92* | Potri.001G118800.1 | PtrMYB92-F | CGAATACTAACGACGACACG |
|  |  | PtrMYB92-R | GTGTTCCATCTCTAATGTGC |
| *PtrMY152* | Potri.017G130300.1 | PtoMY152-F | GAAGACTTGCTACTGCCAGAT |
|  |  | PtoMY152-R | TCATTCTTGAGCACTGATTG |
| *ERF1* | At3g23240 | ERF1-F | ATTCTTTCTCATCCTCTTCTTCT |
|  |  | ERF1-R | CGAATCTCTTATCTCCGCCG |
| *AtERF14* | At1g04370 | AtERF14-F | GGATCAAGGAGGTCGTAGCAGTGG |
|  |  | AtERF14-R | TTATTGCCTCTTGCCCATGTTG |
| *PtERF1* | Potri.010G072300.1 | PtERF1-F | AGTTGCCAAAAGAACTCC |
|  |  | PtERF1-R | CCCATCTCATAATGATGAC |
| *PtERF14* | Potri.010G072400.1 | PtERF14-F | CTCTCTCAAGCGATAGGATC |
|  |  | PtERF14-R | GCTTGCACCAACATCACTA |
